# Optimizing self-pollinated crop breeding employing genomic selection: From schemes to updating training sets

**DOI:** 10.3389/fpls.2022.935885

**Published:** 2022-10-06

**Authors:** Felipe Sabadin, Julio César DoVale, John Damien Platten, Roberto Fritsche-Neto

**Affiliations:** ^1^ School of Plant and Environmental Sciences, Virginia Tech, Blacksburg, VA, United States; ^2^ Department of Crop Science, Federal University of Ceará, Fortaleza, Ceará, Brazil; ^3^ International Rice Research Institute (IRRI), Los Baños, Philippines; ^4^ H. Rouse Caffey Rice Research Station, Louisiana State University (LSU) AgCenter, Rayne, LA, United States

**Keywords:** recurrent genomic selection, training set design, stochastic simulation, self-pollinated crops, GS-based methods

## Abstract

Long-term breeding schemes using genomic selection (GS) can boost the response to selection per year. Although several studies have shown that GS delivers a higher response to selection, only a few analyze which stage GS produces better results and how to update the training population to maintain prediction accuracy. We used stochastic simulation to compare five GS breeding schemes in a self-pollinated long-term breeding program. Also, we evaluated four strategies, using distinct methods and sizes, to update the training set. Finally, regarding breeding schemes, we proposed a new approach using GS to select the best individuals in each F2 progeny, based on genomic estimated breeding values and genetic divergence, to cross them and generate a new recombination event. Our results showed that the best scenario was using GS in F2, followed by the phenotypic selection of new parents in F4. For TS updating, adding new data every cycle (over 768) to update the TS maintains the prediction accuracy at satisfactory levels for more breeding cycles. However, only the last three generations can be kept in the TS, optimizing the genetic relationship between TS and the targeted population and reducing the computing demand and risks. Hence, we believe that our results may help breeders optimize GS in their programs and improve genetic gain in long-term schemes.

## Introduction

Genomic selection (GS) has been successfully employed in plant and animal breeding (Heffner et al., 2011a; Spindel et al., 2015; Wolc et al., 2015; Garner et al., 2016). In this technique, breeders build a training set (TS) containing genotyped and phenotyped individuals to estimate the marker effects and use them to calculate genomic estimated breeding values (GEBV) of only genotyped individuals (Meuwissen et al., 2001). Selecting genotypes based on only genomic information allows one to elect them early, which fastens breeding schemes and increases the genetic gain (Beyene et al., 2015; Voss-Fels et al., 2019). However, several parameters influence the accuracy of GEBV prediction, including TS size (Heffner et al., 2011b; Lorenz, 2013), population structure (Guo et al., 2014), the genetic relationship between the TS and selection candidate genotypes (Lorenz and Smith, 2015), and trait heritability (Combs and Bernardo, 2013).

Several studies have examined the optimal employment of GS in self-pollinated crop breeding programs (Lorenz et al., 2012; He et al., 2016; Michel et al., 2016). The self-pollinated crop breeding programs are composed of three phases: creating genetic variation, identifying desirable recombinant lines within progenies, and stabilizing and advancing the desired genotype (Brown et al., 2014). Several breeding methods increase the homozygosity during the line fixation step, and single-seed descent (SSD) is the method most suited to accelerating a breeding scheme. In rice, especially in the IRRI breeding program, the rapid generation advance (RGA) method, consisting of an SSD method conducted in greenhouses or screenhouses, aims to accelerate and shorten the plant growth cycle (Collard et al., 2017). Thus, considering the breeder’s equation that is based on selection intensity, selection accuracy, additive variance, and cycle time, GS and RGA combination can boost genetic gain in rice long-term breeding schemes, principally due to shortening each cycle, increasing the response to selection (Falconer and Mackay, 1996).

Long-term breeding schemes should be analyzed in terms of genetic gain, response to selection, genetic variance, and GS accuracy. Even though several studies involve GS in long-term recurrent selection schemes (Goddard, 2009; Jannink, 2010; Muleta et al., 2019), only a few analyze what is the best breeding stage for applying it. (Bassi et al., 2016; Gaynor et al., 2017). Mendonça et al. (2020) showed that GS increases the efficiency in advanced breeding phases when using a low-intensity selection for quantitative traits in soybean early phases. Marulanda et al. (2016) verified different strategies to apply GS in hybrid breeding programs in several self-pollinated crops. They concluded that using GS followed by one-stage phenotypic selection produced the highest genetic progress. Hence, there is room to optimize GS employment, considering different strategies and breeding stages to boost genetic gain and improve efficiency in a breeding program.

Another point to highlight in long-term breeding schemes employing GS is that prediction accuracy (PA) declines over cycles (Jannink, 2010; Muleta et al., 2019). This phenomenon might be due to the reduction of the genetic relationship between TS and selection candidate genotypes (Lorenz and Smith, 2015), the breakdown of linkage disequilibrium (LD) between marker and QTL by recombination (Jannink, 2010; Müller et al., 2017), and genetic variability decreasing in the population. In this context, few studies shed light on TS updating requirements (Jannink, 2010; Muleta et al., 2019; DoVale et al., 2021) to reduce the PA decline across breeding cycles. There are several manners to select individuals to update TS population, and selecting them based on genetic relationship with candidate genotypes to selection seems to be the best strategy to minimize the PA decline (Neyhart et al., 2017). Therefore, our study aimed to optimize the use of GS and identify a method to update the TS with greater efficiency in self-pollinated crop breeding programs. Also, we proposed a new method employing GS to select the best individuals in each F2 progeny to cross them and generate a new recombination event.

## Material and methods

Our study compared different GS strategies applied in a long-term breeding program. For that, we used rice (*Oryza sativa* L.) as a self-pollinated model crop and stochastic simulations performed by the *AlphaSimR* package (Gaynor et al., 2021). Furthermore, we evaluated different strategies for updating the TS and how it influences genetic parameters over breeding cycles.

### Long-term breeding schemes employing genomic selection

#### Historical population and genetic parameters

A historical rice founder population was simulated as 1,000 unique diploid inbred individuals, with 12 chromosome pairs each, using a Markovian Coalescent Simulator (MaCS) (Chen et al., 2009). For that, 1,644 biallelic segregating sites were considered, uniformly distributed across chromosomes and 360 segregating loci randomly sampled as quantitative trait nucleotides (QTN), and 994 segregating loci as single-nucleotide polymorphism (SNP). The genome size (cM) and chromosome sizes follow those values described by Li et al. (2008).

In order to simulate a quantitative trait as yield, we used the genetic parameters obtained by Li et al. (2008). Each QTN received randomly additive and dominance effects. Genetic values for each genotype were obtained by summing all additive and dominance effects for all QTN. Additive effects (*a*) were sampled of a gamma distribution with scale and shape parameters equal to 1 and randomly assigned for each QTN. Similarly, dominance effects (*d*) for each QTN were computed by multiplying the absolute value of its additive effect (*a_i_*) by locus-specific dominance degree (*δ_i_* ​). Dominance degrees were sampled of a Gaussian distribution with $\delta_{i}\sim N(\mu_{\delta },\sigma_{\delta }^{2})$ , where *μ_δ_* is the average dominance degree equal to 0.22 and $\sigma_{\delta }^{2}$ is the dominance variance equal to 0.50. Finally, dominance effects were assigned for each QTN according to the equation below:


$$d_{i}=\{\begin{array}{c} 0,\;\;if\;QTN\;is\;homozygous\\ \delta_{i}\times |a_{i}|,\;\;if\;QTN\;is\;heterozygous \end{array}$$

Phenotypic values were obtained by adding a random error sampled of a Gaussian distribution with mean equal to 0 and variance ( $\sigma_{e}^{2}$ ) equal to 1, which was defined by broad-sense (*H* ^2^ = 0.53) and narrow (*h* ^2^ = 0.50) heritabilities.

#### Base population and burn-in phase

In order to obtain our base population, we selected 48 individuals based on their superior phenotypic values from 1,000 lines of the historical population. As a starting point to consider a representative program as current 4-year rice breeding programs, we simulated five traditional recurrent selection cycles totaling 20 years of breeding in the burn-in stage. These 48 parental lines were crossed to generate 30 F_1_ plants, which were selfed to produce 230 F_2_ plants from each cross (Cobb et al., 2019). SSD was used in line fixation stages until the F_6_, where the best individuals were selected based on their phenotypic values to find the next breeding cycle. After five recurrent breeding cycles, we obtained the base population to evaluate the recurrent genomic selection breeding schemes.

Finally, the base TS was composed of 1,536 inbred lines originated from 30 crosses, between 48 individuals (parents), with nearly 52 plants per cross, from the base population after the burn-in stage. Markers effects were predicted using the ridge-regression best linear unbiased prediction (RRBLUP) (Endelman, 2011) according to the equation below:


$$y=1\mu +\;Z_{u}u+\;\epsilon$$

where ***y*** is the vector of individual phenotypic values from the TS; *μ* is the mean (intercept); ***u*** is the vector of marker effects, where $u\;\sim \;N(0,I\sigma_{u}^{2})$ ; and ***ϵ*** is the vector of random residuals. **1** is the vector of ones and ***Z_u_*** is the incidence matrix of TS genotypes for *m* markers. ***Z_u_*** is coded as 1 for homozygous A_1_A_1_, -1 for homozygous A_2_A_2_, and 0 for heterozygous A_1_A_2_.

To perform the GS, the genomic estimated breeding value (GEBV) was estimated using the following equation: ***GEBV* = *Mu***, where ***M*** is the incidence matrix of selection candidate genotypes, and *u* is the vector of predicted marker effects.

#### Breeding schemes simulations

**Figure 1:**
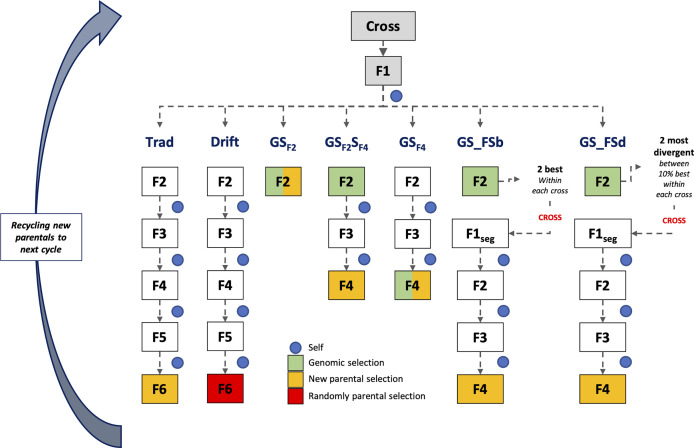
Scheme of the first cycle of the long-term breeding schemes. At the end of each scheme, a parental group was selected for recycling and compounding the next cycle that works independently. Trad: traditional phenotypic selection; Drift: random selection; GS_F2_: genomic selection performed in F2 stage; GS_F2_S_F4_: genomic selection performed in F2 stage and phenotypic selection performed in F4 stage; GS_F4_: genomic selection performed in F4 stage; GS_FSb: genomic selection performed on F2 stage to select the two best individuals based on genomic estimated breeding values and crossed them to make a new recombination event; GS_FSd: genomic selection performed on F2 stage to select the two most divergent individuals and crossed them to make a new recombination event.

Simulations were based on the rice breeding program structure from the International Rice Research Institute (IRRI) (Collard et al., 2019). For all scenarios, the line fixation phase was conducted by the single-seed descent (SSD) method, which collects one seed from each segregating plant to advance to the next stage until it reaches a high homozygosity level. Seven breeding schemes were compared, with different timelines and GS procedures (**Figure 1**). Five schemes employed GS in distinct stages during the line fixation phase, and two phenotypic breeding schemes (traditional and drift) were used as baselines. Additionally, two group sizes of parental lines (24 and 48) were used to populate the crossing block, totaling 14 different scenarios. The parental line group size aims to analyze the effect of selection intensity over the coming cycles and their consequences on the breeding population’s performance and genetic variability. In this case, we make a naive assumption, considering the number of parents is a proxy for the effective population size (Ne).

For all scenarios (breeding schemes and population sizes), we used the same base population aforementioned. In the first cycle, all breeding schemes were the same from crossing to the F_2_ generation. Thirty crosses were randomly made from the parental lines. Then, F_1_ plants were selfed to produce an F_2_ segregating generation with 230 F_2_ plants per cross (Cobb et al., 2019). From the F_2_ phase, the different long-term breeding schemes were applied until the new parental group selection for the next cycle. After the second cycle, each scheme followed independently and was simulated over 20 breeding cycles and 100 replicates, totaling 2,000 estimates (**Figure 1**).

Traditional (Trad) and drift (Drift) schemes were conducted by the SSD method from F2 to F6 generation. Each generation lasted about 180 days, totaling four years for each cycle. These two schemes only differed on parental group selection: the best phenotypic values for Trad and a random sample for Drift. The line fixation phase for breeding schemes using GS was conducted by the rapid generation advanced (RGA) method. The RGA method is an SSD method carried out in greenhouses or screenhouses to accelerate and shorten the growth plant cycle (~ 90-100 days) (Collard et al., 2017). All breeding schemes using GS employed the TS composed by 1,536 inbreed lines, as aforementioned. Below are details about each breeding scheme:

- GS_F2_: GS was performed in the F_2_ stage, and all plants were genotyped. Individuals with the highest GEBV in F_2_ were selected (24 or 48), regardless of progenies, to compound the next cycle (one year per cycle).
- GS_F2_S_F4_: GS was performed in the F_2_ stage, and genotypes with the highest GEBV within each progeny were selected for the next generation (F_3_). So, 230 F_3_ plants from the selected F_2_ plants were conducted until F_4_. Then, in F_4_, individuals with the highest phenotypic values were selected, regardless of progenies, to compound the next cycle (2 years per cycle).
- GS_F4_: GS was performed in the F_4_ stage, and all plants were genotyped. Individuals with the highest GEBV in F_4_ were selected (24 or 48), regardless of progenies, to compound the next cycle (2 years per cycle).
- GS_FSb: GS was performed in the F_2_ stage, and the two individuals with the highest GEBV within each progeny were selected and crossed to generate 230 F_1seg_ segregating plants. These plants were conducted until F_4_, and individuals with the highest phenotypic values were selected, regardless of progenies, to compound the next cycle (2.33 years per cycle).
- GS_FSd: GS was performed in the F_2_ stage, and the highest individuals (10%) based on GEBV were selected to calculate the Euclidean genetic distance between them. The most divergent sibs were crossed within each progeny to generate 230 F_1seg_ segregating plants. These plants were conducted until F_4_, and individuals with the highest phenotypic values were selected, regardless of progenies, to compound the next cycle (2.33 years per cycle).

Theveragee genetic value, the genetic value of the best genotype, additive genetic variance, and PA were calculated for each breeding cycle. The PA was calculated as the Pearson correlation between true genetic values and GEBV. For the Trad scheme, PA was computed as the square root of heritability (*h* ^2^), whereas for the Drift scheme as considered zero due to random selection. Furthermore, the response to selection after ten years of breeding was calculated following the equation:


$$RS=\frac{X_{i}-X_{0}}{t}$$

where *RS* is the relative response to selection to the first breeding cycle; *X_i_* is the genetic value mean of the parental group in the *i* cycle; *X*_0_ is the genetic value mean of the base population, and *t* is the breeding cycle time, in this case, ten years.

### Training sets update scenarios

Different strategies examined the effect of TS updating on the breeding schemes. The crossing block was fixed at 48 parental lines for each cycle, and the GS_F2_S_F4_ scheme was used as the breeding scheme. In addition, Trad and Drift schemes were used as benchmarks. Below are details about the updating scenarios:

- TS_C0_: the TS was composed of 1,536 individuals without updates. Markers effects were used to predict genetic values and select genotypes in future cycles.
- TS_CN_: TS was initially composed of 384 individuals. For each new cycle, a new TS was built with 13 random individuals from each F_4_ progeny, totaling 384 individuals. Marker effects were estimated in each cycle and used to predict genetic values and select genotypes in the next cycle.
- TS_ALL_: TS was initially composed of 384 individuals. Every cycle, new individuals were added to TS (13 from each F_4_ progeny), increasing TS size by 384 each cycle (384, 768, 1152, …, 7,680). Marker effects were estimated in each cycle and used to predict genetic values and select genotypes in the next cycle.
- TS_GPO_: TS was composed of three sets of 384 individuals, one set per generation (grandparents, parents, and offspring). Every cycle, the newest set (13 randomly individuals from each F_4_ progeny) was included, and the oldest one was removed. Markers effects were estimated in each cycle and used to predict genetic values and select genotypes in the next cycle.

Finally, only for the TS_GPO_ scenario, three different TS sizes (384, 768, and 1152) were simulated to add into the TS every breeding cycle, aiming to recalibrate the marker effects and keep the GS accuracy at satisfactory levels for more generations. Each TS updating strategy was simulated over 20 breeding cycles and replicated 100 times. Similar to the breeding schemes, PA was calculated for each cycle and replicate.

## Results

### Influence of the breeding schemes and number of parental lines

**Figure 2:**
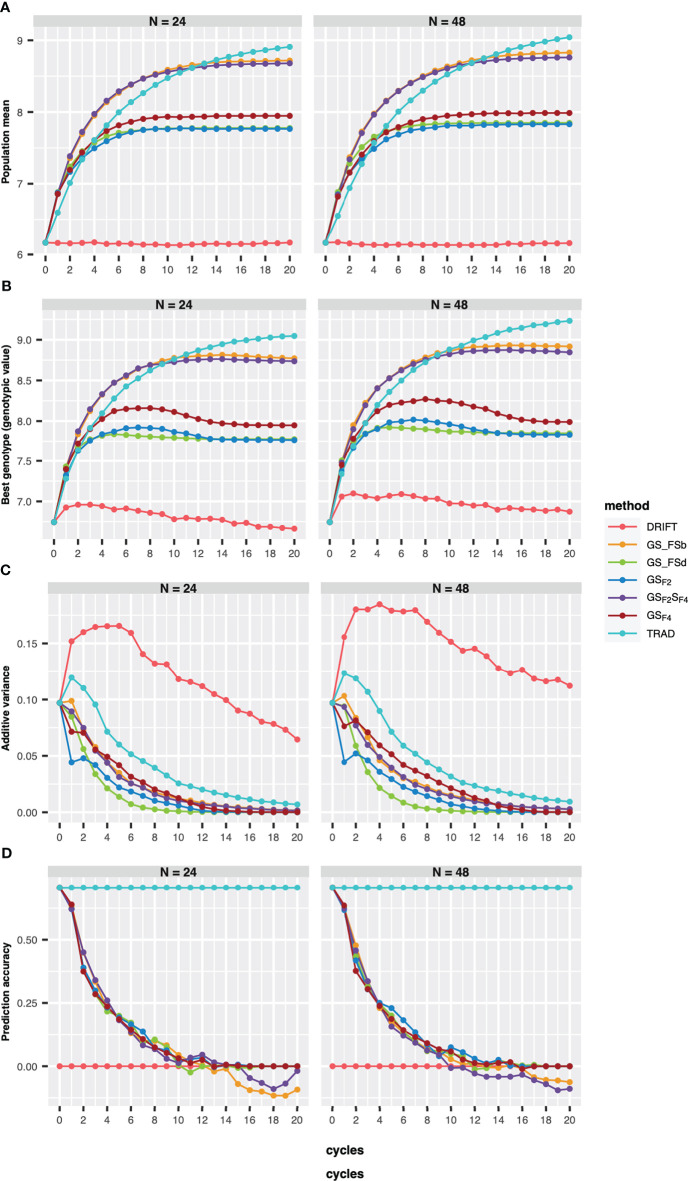
Population mean **(A)**, the best genotype **(B)**, additive variance **(C)**, and prediction accuracy of selection **(D)** over 20 recurrent cycles using two sizes of parental lines (N=24 and N=48). Each colored line represents a breeding scheme. Trad: traditional phenotypic selection; Drift: random selection; GS_F2_: genomic selection performed in F2 stage; GS_F2_S_F4_: genomic selection performed in F2 stage and phenotypic selection performed in F4 stage; GS_F4_: genomic selection performed in F4 stage; GS_FSb: genomic selection performed on F2 stage to select the two best individuals based on genomic estimated breeding values and crossed them to make a new recombination event; GS_FSd: genomic selection performed on F2 stage to select the two most divergent individuals and crossed them to make a new recombination event.

Regarding the number of parental lines, GS-based methods showed similar performances for the population mean (genetic mean), the best genotype performance, PA, and additive genetic variance (**Figures 2A, B**). However, for the Trad scheme, the population mean plateau was reached earlier using 24 than 48 parental lines., whereas GS_FSb and GS_F2_S_F4_ showed the best performance for GS-based methods for both parental sizes. Furthermore, GS_FSb and GS_F2_S_F4_ revealed higher population mean and the best genotype performance in the earliest breeding cycles (**Figure 2**).

Our results revealed a reduction of additive variance across breeding cycles for all schemes (**Figure 2C**). Using 48 parental lines resulted in slightly higher additive variance values, and the drift effect was more pronounced on genetic variability when using 24 parents (**Figure 2C**). Moreover, all GS breeding schemes revealed a PA reduction across the cycles (**Figure 2D**), reaching negative values from the eighth cycle, for 24 parents, whereas using 48 parentals, only GS_FSd and GS_FSb showed negative values from the eighth and tenth cycle, respectively.

**Figure 3:**
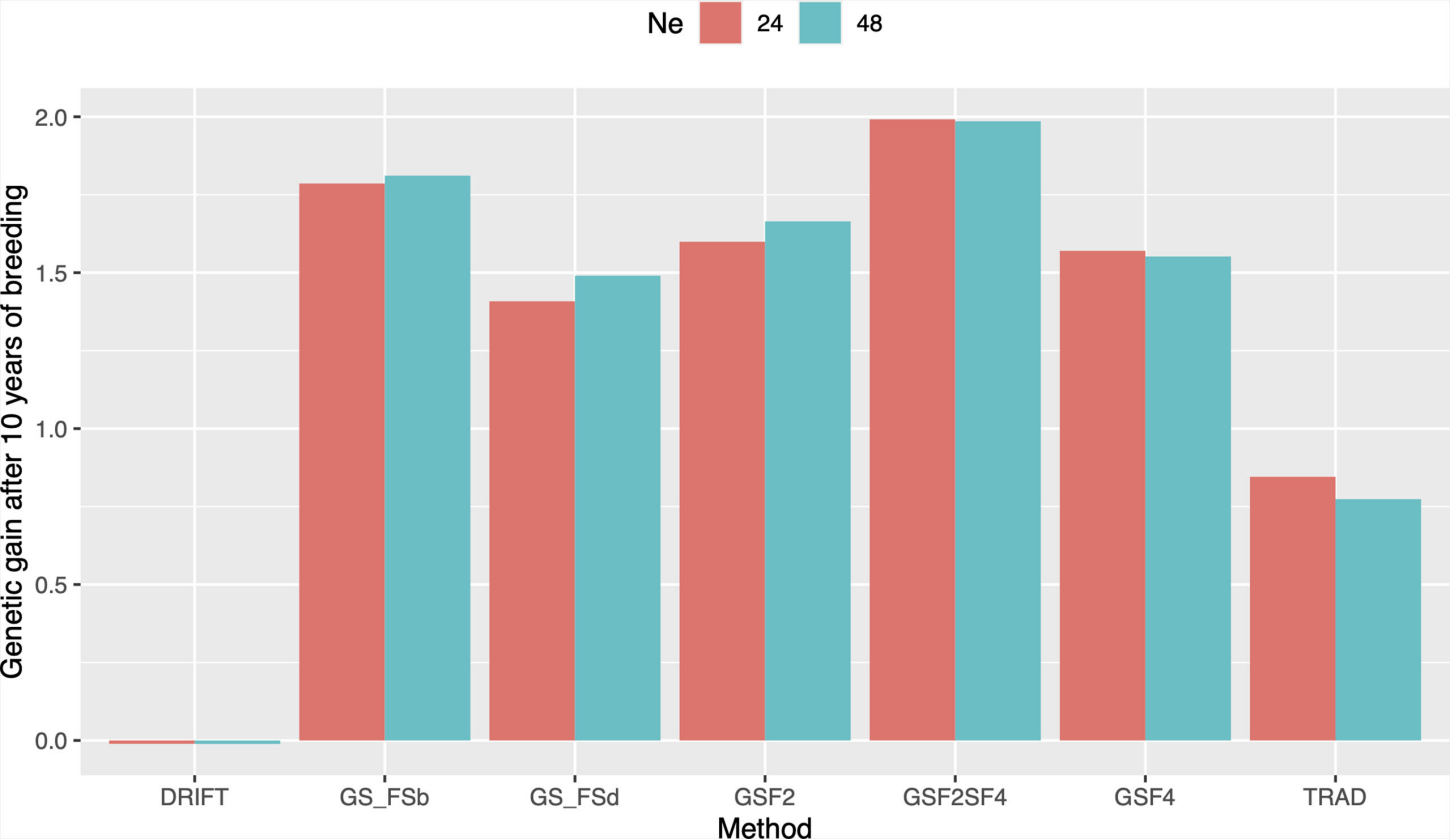
Genetic gains after ten years of breeding using different selection schemes and two sizes of parental lines (N=24 and N=48). Trad: traditional phenotypic selection; Drift: random selection; GS_F2_: genomic selection performed in F2 stage; GS_F2_S_F4_: genomic selection performed in F2 stage and phenotypic selection performed in F4 stage; GS_F4_: genomic selection performed in F4 stage; GS_FSb: genomic selection performed on F2 stage to select the two best individuals based on genomic estimated breeding values and crossed them to make a new recombination event; GS_FSd: genomic selection performed on F2 stage to select the two most divergent individuals and crossed them to make a new recombination event.

In order to evaluate response to selection, we defined ten years of breeding horizon to place all schemes on the same page since they have different lengths. By doing so, GS_F2_S_F4_ method outperformed the others, showing a response to selection 166% higher than the Trad scheme (**Figure 3**). Moreover, GS_F2_S_F4_ revealed a genetic gain 20% higher than fast recycling methods, such as GS_F2_, highlighting the trade-off between the number of cycles and the importance of updating the training sets. Furthermore, using 24 parents was slightly better than 48, but, from our perspective, it does not compensate for the risks due to the drift effect. Hence, we considered 48 parents and the GS_F2_S_F4_ breeding scheme for further comparisons.

### Effect of training set update

**Figure 4:**
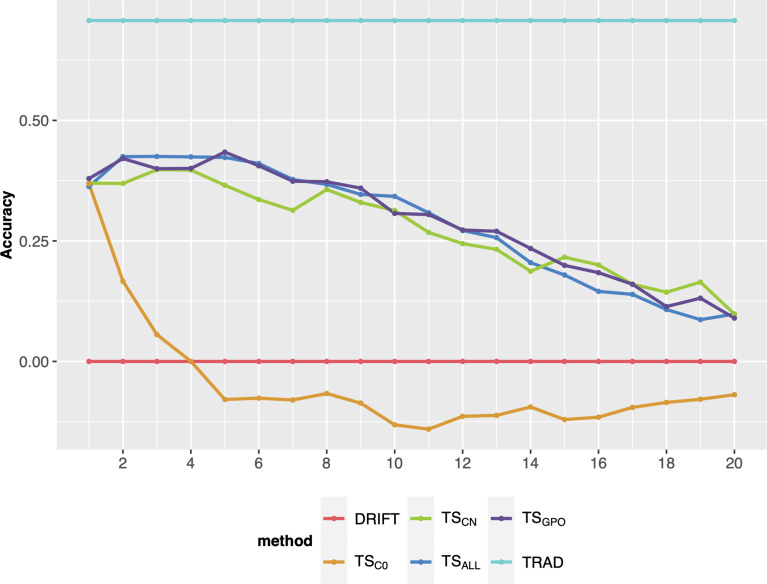
Prediction accuracy of selection over 20 breeding cycles *via* GS_F2_S_F4_ scheme considering 48 parental lines using different methods to update the training set. Each colored line represents an updating training set method. TS_C0_: training set without updates; TS_CN_: a new training set was built every cycle with the previous generation; TS_ALL_: training set updated every cycle with the addition of a new set from the previous cycle; TS_GPO_: training set built with the three last generations and updated each cycle.

After the breeding scheme evaluation, different ways to update the TS were evaluated on their performance on long-term breeding schemes. Overall, the updating methods (TS_CN_, TS_ALL_, and TS_GPO_) produced similar responses in terms of PA over cycles and outperformed the TS_C0_ (no updates). Also, TS_C0_ presented a steep decline in the earliest breeding cycles (**Figure 4**). Differences were negligible among the updated scenarios, so TS_GPO_ was considered the best due to its ease of implementation and stability trend over cycles. Another advantage is, that if for any reason, experiments were missed in a certain year and precluded TS updating, TS_GPO_ might buffer and keep the accuracies at higher levels due to its size. Moreover, the last three generations maximize the genetic relationship between TS and the targeted population and reduce the computing demand due to its affordable size. Therefore, the TS_GPO_ scenario for updating TS was considered for the final comparisons.

**Figure 5:**
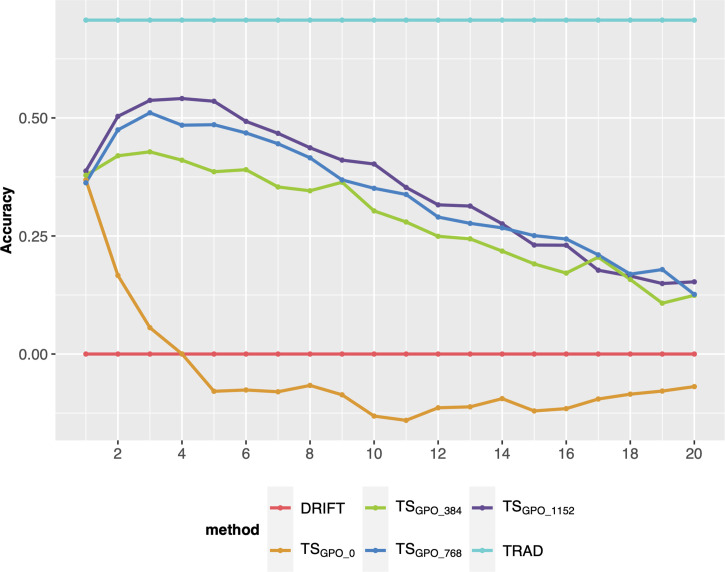
Prediction accuracy of selection over 20 breeding cycles *via* GS_F2_S_F4_ scheme considering 48 parental lines, the TS_GPO_ method, and different sample sizes to update every cycle’s training set. Each colored line represents an updating training sample size. TS_GPO_: training set built with the three last generations and updated each cycle; 0, 384, 768, and 1152 are the number of individuals used to update the TS.

Finally, we compared different sample sizes to update the TS every breeding cycle. Overall, adding sample sizes of 768 (~10% of the breeding program population size after RGA) or more every cycle, keeps the GS accuracy at satisfactory levels for more cycles (up 8 or 10), outperforming the TS_C0_ (no updates) or small sample sizes, such as 384 (**Figure 5**). However, after 8 or 10 cycles, there is a steady decay even for the best methods.

## Discussion

### Breeding schemes and the number of parental lines

GS can speed up breeding schemes and increase genetic gain (Jannink et al., 2010; Varshney et al., 2017). However, to maximize the breeding program efficiency, deciding how and at which stage to apply GS is essential. This study tested different long-term breeding strategies for implemented GS based on a simulated rice breeding program. Furthermore, a new approach is proposed for using GS during the line fixation stage. In this approach, two individuals (best ones - GS_FSb, and most divergent - GS_FSd) within each F_2_ progeny are identified and crossed to generate a new recombination event. These progenies are then fixed using the RGA method until F_4_ to select new parents for the next recurrent cycle.

Our results showed that the number of parental lines to compose the crossing block influences all genetic parameters over breeding cycles. This influence was expected due to the relationship between the number of parental lines, the effective population size (Ne), and the selection intensity. In the scenarios examined here, the number of crosses (crosses = 30) was the same in both scenarios (24 and 48 parentals). However, fewer parents increase the number of crosses with the same parent, which reduces the final Ne and, consequently, the genetic variance. Also, a smaller Ne increases the effect caused by the drift (Hartl and Clark, 2006), reducing genetic variance in long-term breeding cycles. Therefore, the Drift scheme acted as one of the benchmarks for this purpose. In the Drift scheme, using 24 parentals resulted in an additive genetic variance reduction over breeding cycles, whereas using 48 parental lines showed higher resilience to this effect (**Figure 2C**). Furthermore, Ne is a crucial factor for breeders as programs with a smaller Ne will become inbred faster and show no further response to selection (Cobb et al., 2019).

Several studies reported an increase in response to selection in breeding schemes that use GS (Gorjanc et al., 2018; Muleta et al., 2019). In our study, long-term breeding schemes employing GS showed values between 200% (GS_F2_) and 608% (GS_FSd) higher than phenotypic selection (Trad) in the first breeding cycle for response to selection per year (**Figure 2C**). This increase was principally due to the shortening of the breeding cycle with the GS implementation. However, as the breeding cycles progress, they rapidly decreased their response to selection. In addition, GS can accelerate inbreeding by selecting closely related individuals, yet, on the other way, this can also cause a faster exhaustion of the genetic variance faster than phenotypic selection (Jannink, 2010). Our results also revealed a higher decline in genetic variance under GS breeding schemes than a traditional breeding scheme, and consequently, their response to selection decay faster (**Figure 2**). However, this effect can be mitigated and offset by preserving a larger Ne (48 parent scenario), reducing the genetic variance consumed by GS over breeding cycles, and permitting long-term genetic gain.

Genomic selection also increases genetic gain by shortening the breeding cycle to select the best genotypes with phenotyping (Jannink et al., 2010; Lorenz et al., 2011; Crossa et al., 2017). However, breeders need to consider the mode of reproduction of the species associated with the breeding method when deciding to apply GS to reach the desirable effect (Brown et al., 2014). For example, using early generations (F4 or early) instead of doubled haploid lines can hinder the selection of the best new parentals once the residual heterozygosity reduces the precision of estimating QTL effects, the additive genetic variance, and consequently, the PA. This fact can be more prominent if the trait is controlled by several QTLs and has low heritability (Mayor and Bernardo, 2009). However, residual heterozygosity tends to be lower than expected due to the increase in inbreeding over recurrent selection cycles in a closed system, crossing only elite by elite parents. Also, rice does not currently have scalable and feasible procedures to generate doubled-haploid populations, hence, in this study, the RGA method was used to advance generations and increase endogamy in our GS breeding schemes. This method reduces the rice growth cycle to 90-100 days, depending on the genotype (Collard et al., 2017). Differences in the number of generations in the breeding schemes examined led to differences in the total cycle time. For instance, the Trad scheme takes four years to complete each cycle, whereas the GS_F2_ only takes six months. The GS_F2_S_4_ yielded 166% higher genetic gain than the Trad scheme and 20% better than GS_F2_ in ten years of breeding (**Figure 3**). Another interesting point is that the best scenarios are composed of 2-stages selection. This result shows that besides longer, 2-stages combining GS and phenotypic selection compensates rather than applying rapid cycling, using only GS at stage-1 for recycling parents. Therefore, our results reinforce that to obtain sustainable long-term genetic gain in long-term breeding schemes is crucial to consider a balance between genetic gain, PA, time, and genetic variance over the breeding cycles (Jannink et al., 2010). In this context, some questions arise for further studies: do we get progressive increases in effectiveness with 3, 4, and 5 stages of selection? E.g., GS at F2 + GS at F4 + Phenotypic at F6? Do we need different types of selection (we used GS and phenotypic here - 2 different types)? Would the same response be shown with, e.g., two rounds of GS?

Few studies explore the best phase or stage to apply GS to maximize the genetic gain in long-term breeding schemes (Gorjanc et al., 2018; Bassi et al., 2016). A new method employed GS in the line fixation stage in a rice breeding program. In this scheme, a cross was simulated between two individuals selected by GS in the F_2_ generation considering the best GEBVs (GS_FSb) and the most divergent between best GEBVs (GS_FSd). Among the GS-based schemes, the GS_FSb reached the highest population mean over the breeding cycles (**Figure 2A**). The strategy of crossing the best individuals based on GEBV probably enriched the parents in which major-effect alleles were fixed since these individuals may have the same genotype for these alleles (Zhong et al., 2009; Jannink, 2010). Also, loci with minor effects, but important for the trait could be divergent between these individuals, and recombination between these loci may increase the probability of a future line having this favorable allele in homozygosity. On the other hand, when we crossed the most divergent individuals, major loci may have different alleles in each genotype. Therefore, the probability of achieving a future line with the favorable allele in this locus is lower, and indeed GS_FSd had one of the worst results considering genetic value over breeding cycles.

Even though GS_FSb has shown the highest population mean over breeding cycles, its values were close to the GS_F2_S_F4_ scheme. However, further considering genetic gain, it is crucial to regard the practicality of realizing each breeding scheme in real conditions. Making a new cross between two individuals from an F_2_ progeny brings additional labor and costs. Hence, since the difference between GS_FSb and GS_F2_S_F4_ is negligible, we consider the GS_F2_S_F4_ the best scenario to employ GS.

### Effect of training set update strategies

The second objective of this study was to identify a better strategy for TS updating to guarantee a higher genetic gain and avoid the decline of accuracy across long-term breeding cycles. As mentioned, all genomic breeding schemes showed a reduction of accuracy over breeding cycles (**Figure 2D**). After the first breeding cycle, PA showed a substantial decrease, and by the last breeding cycles, GS was practically by chance, with PA close to zero. This situation can hinder the selection of the best genotypes, and consequently, the population’s genetic performance does not increase and may sometimes decrease (Jannink, 2010; Neyhart et al., 2017). This observation is consistent with several other studies showing the PA reduction in long-term breeding schemes (Jannink, 2010; Müller et al., 2017; Muleta et al., 2019). Working with GS optimization in a sorghum breeding program, Muleta et al. (2019) verified that the PA declined over breeding cycles, especially for oligogenic traits. The latter study also showed that TS updating slowed the decline in accuracy over breeding cycles but did not prevent this entirely. In our results, all scenarios involving GS with TS updating reached the best population performance compared to the phenotypic selection, at least until the sixth breeding cycle. TS_C0_ had the highest population mean values in initial cycles, which shows that a larger TS delivers a higher accuracy (Cericola et al., 2017; Norman et al., 2018) due to a larger number of allelic observations is required to predict small QTL effects accurately (Gilmour, 2007). However, the TS_C0_ displayed a higher decrease in PA across breeding cycles since this scenario did not update the TS (**Figure 4**). After the fourth cycle, its PA reduction may promote a lower population performance than other updating scenarios due to the lack of reliability in the selection process. Amongst scenarios that did include TS updating, the TS_N_ scenario was more unstable than TS_GPO_ or TS_ALL_. These results show that it is crucial to maintain a high genetic relationship between the TS and selection candidate genotypes when updating TS.

Another question about updating the TS is which lines and the number of lines to select. Neyhart et al. (2017) studied updating TS methods in a barley long-term breeding program. Updating the TS minimizes the decline in accuracy due to updating LD between markers and QTL. Furthermore, it was found that the PA was slightly higher when the TS contained only the most recent data, whereas adding the best individuals from each cycle in long-term breeding schemes resulted in the highest genetic gain (Neyhart et al., 2017).

All updating methods avoided an abrupt decline in PA across breeding cycles, corroborating with previous studies (Neyhart et al., 2017; Muleta et al., 2019). In the first cycle, TS_C0_ PA presented the highest accuracy (0.40), which the TS size effect can explain (Müller et al., 2017; Zhang et al., 2017; Norman et al., 2018). However, TS_C0_ PA over breeding cycles presented the highest decline displaying negative values from the eighth cycle. This is because the TS_C0_ scenario did not update the training set, and selection candidate genotypes were selected only using the initial marker effects. Hence, the genetic relationship between the TS and selection candidate genotypes decreased across breeding cycles, resulting in PA decline (Lorenz and Smith, 2015).

When considering the maintenance of PA and genetic gain across breeding cycles, it requires maintaining a balance between the genetic relationship between the TS and selection candidate genotypes and accurate estimates of LD between markers and QTL. However, in long-term recurrent schemes, new recombination events occur at each breeding cycle, which causes a breakdown of LD between markers and QTL, consequently decreasing PA (Jannink, 2010; Müller et al., 2017). High-density marker panels can reduce an abrupt PA decline across breeding cycles and, therefore, deliver higher genetic gain in long-term genomic recurrent schemes (DoVale et al., 2021). Our simulation scenarios used a low-density marker panel, which may explain a lower PA resulting from a lower probability of LD between the marker and the QTL, finishing in a smaller fraction of explained genetic variation (Solberg et al., 2008). Hence, high-density marker panels could help increase the probability of finding markers in LD with the same QTL across different cycles (Daetwyler et al., 2010).

This study used an SNP chip containing 1,000 markers to simulate the SNP panel optimized for the IRRI irrigated breeding program (Arbelaez et al., 2019). Low-density SNP panels are attractive for GS due to their cost-effectiveness (Vallejo et al., 2018; Arbelaez et al., 2019; Al-Tobasei et al., 2021). In this context, our results showed that updating the TS with an affordable size reduced the PA decline over breeding cycles, presenting a lower decay than in other studies (Neyhart et al., 2017; Muleta et al., 2019), even using a low-density marker set. Furthermore, updating the TS promoted more accurate estimates of LD between markers and QTL since, in long-term breeding schemes, recombination between marker and QTL causes an LD decrease, whereas selection and drift act to generate new LD or tighten the LD between closely linked loci (Hill and Robertson, 1968; Habier et al., 2007; Lorenz et al., 2011). This outcome is crucial as SNP chips with few markers may permit breeding programs with limited resources to use GS in their pipeline. However, it seems not essential to consider previous cycles to estimate the LD patterns between markers and QTL since TS_ALL_ and TS_GPO_ were similar to those obtained *via* TS_CN_, which counts only the last breeding cycle. However, as described earlier, we considered TS_GPO_ a straightforward and stable method. Furthermore, it might buffer and maintain the accuracies at good levels in cases of missing a year of trials.

Finally, even with updating the training set, the accuracy still tends to reduce drastically to very low levels after 10-12 cycles. The main explanation is the lack of genetic variability due to the high intensity and closed related parents’ selection. For both, we can monitor the genetic variability over the cycles and, at the recombination step, include external parents into the crossing block. Also, we could define restrictions in the parental selection, optimizing the trade-off between response to selection and relatedness, for instance, setting a maximum number of parents from each cross.

## Conclusion

Implementation of genomic selection on long-term breeding schemes may accelerate genetic gains. However, it is crucial to determine at and when to implement the genomic selection since it can produce lower genetic gain than phenotypic selection, depending on the strategy. For example, applying genomic selection in an F_2_ progeny followed by a phenotypic selection of new parentals in F_4_ produced the highest genetic gain across breeding cycles. Moreover, updating the training set allowed better maintenance of prediction accuracy over recurrent breeding cycles. Adding a new and proper amount of information (over 768 individuals) every cycle into the training set allows re-estimation of the marker’s effects. In other words, updating the LD between markers and underlying QTL guarantees the highest genetic gain over recurrent selection cycles. Finally, only the last three generations should be kept in the TS, optimizing the genetic relationship between TS and the targeted population in a closed system.

## Data availability statement

The original contributions presented in the study are included in the article/**Supplementary Material**. Further inquiries can be directed to the corresponding author.

## Author contributions

FS wrote the manuscript, discussion, contributing to ideas and graphs. JD and JP contributed to the writing, mainly discussion. RF-N elaborated on the hypothesis, conducted the analyses, interpreted the results, and contributed to the writing. All authors contributed to the article and approved the submitted version.

## Funding

AGGRi Alliance (Accelerated Genetic Gain in Rice in South Asia and Africa - OPP1194889) - Bill and Melinda Gates Foundation (BMGF).

## Acknowledgments

To Dr. Gary Atlin (Bill and Melinda Gates Foundation), IRRI Breeders, Excellence in Breeding - CGIAR platform, and Allogamous Breeding lab-USP members for the comments and suggestions.

## Conflict of interest

The authors declare that the research was conducted in the absence of any commercial or financial relationships that could be construed as a potential conflict of interest.

## Publisher’s note

All claims expressed in this article are solely those of the authors and do not necessarily represent those of their affiliated organizations, or those of the publisher, the editors and the reviewers. Any product that may be evaluated in this article, or claim that may be made by its manufacturer, is not guaranteed or endorsed by the publisher.
