# Supplementary material for: Optimizing self-pollinated crop breeding employing genomic selection: From schemes to updating training sets
Source: Front Plant Sci. 2022 Oct 6;13:935885. doi: 10.3389/fpls.2022.935885 (PMC9583387; doi:10.3389/fpls.2022.935885)
Supplement: Supplementary file 1 [file DataSheet_1.pdf]

```
###########################################
#  Creating and comparing breeding schemes
###########################################
(start.time <- Sys.time())
require(AlphaSimR)
require(foreach)
require(doParallel)
#################### Parameters #############
progenie.size <- 230
nQTL <- 360
h2 <- 0.50
H2 <- 0.53
add <- 0.22
segSites <- 1644
chip.size.1 <- 996
replicates <- 100
r <- 1:replicates
cycles <- 20
nThreads <- 4
n.parents <- 48
n.crosses <- 30

# setting the number of cores that will be used
registerDoParallel(cores = nThreads) # type the number of cores you
want to use
getDoParWorkers()

###########################
# crop history of evolution
###########################
set.seed(29121983)

history <- runMacs(nInd = 1000,
                   nChr = 12,
                   segSites = segSites / 12,
                   inbred = TRUE,
                   species = "GENERIC",
                   split = NULL,
                   ploidy = 2L)

# then, add the simulation parameters to the simulated population
SP = SimParam$new(history)

# Lets add a trait, considering gamma distribution for QTL effect;
A + D effects
SP$restrSegSites(nQTL/12, chip.size.1/12)
SP$addTraitAD(nQtlPerChr = nQTL / 12, gamma = TRUE, mean = 0, var =
1, meanDD = add, varDD = 0.5)

# setting the heritability of the trait, narrow and broad sense
SP$setVarE(h2 = h2, H2 = H2)

# add a SNP chip to our population
SP$addSnpChip(chip.size.1/12)
```

```
# after the historical population (mimic the crop evolution, it is
time to simulate the first years of a breeding program, like as a
warm up process)
firstPop <- newPop(rawPop = history, isDH = TRUE, simParam = SP)

#########################################################
# simulating the founders
#########################################################
# select the first parents, full inbreed
set.seed(29121983)
founders <- selectInd(firstPop, nInd = n.parents, trait = 1, use =
"pheno", gender = "B",
                 selectTop = TRUE, returnPop = TRUE, candidates =
NULL,
                 simParam = SP)

#############################################################
# simulating 5 generations of rice trad breeding (SSD) = burn-in
#############################################################
cat("Burn-in", "\n")

pop.trad <- founders
perform <- c()

for (i in 1:5) {
  cat("Processing the cycle", i, "\n")
  #F1 and F2
    f1 <- randCross(pop = pop.trad, nCrosses = n.crosses, nProgeny =
1, simParam = SP)
    f2 <- self(pop = f1, nProgeny = progenie.size, simParam = SP)

  #F2 to F6 by SSD
    fn <- f2
  for (j in 1:4) {
      fn <- self(pop = fn, nProgeny = 1, simParam = SP)
  }

  # then, select the best one regardless the family
  newparents <- selectInd(pop = fn, nInd = n.parents, trait = 1,
"pheno", gender = "B", selectTop = TRUE, simParam = SP)
  pop.trad <- newparents
  perform <- c(perform, meanG(pop.trad))
}


#########################
# creating the first and small TS for the different ways of update
#  a population of 384 - one chip
#########################
cat("TS", "\n")
#cross everyone
TS0 <- randCross(pop = pop.trad, nCrosses = n.crosses, nProgeny =
13, simParam = SP)[1:384]
```

```
# recombining the offspring
TS00 <- newPop(rawPop = TS0, simParam = SP)

# obtaining full inbred lines by DH tech
TS000 <- makeDH(pop = TS00, nDH = 1, useFemale = TRUE, simParam =
SP)

markers <- RRBLUP(pop = TS000, traits = 1, use = "pheno", snpChip =
1, simParam = SP)


#########################
# creating a specific TS for scenario C0
# a population of 1536 - 4 chips 0f 384
#########################

#cross everyone
TS <- randCross(pop = pop.trad, nCrosses = n.crosses, nProgeny = 52,
balance = TRUE,
                parents = NULL, simParam = SP)[1:1536]

# recombining the offspring
TS1 <- newPop(rawPop = TS, simParam = SP)

# obtaining full inbred lines by DH tech
TS2 <- makeDH(pop = TS1, nDH = 1, useFemale = TRUE, simParam = SP)

markersC0 <- RRBLUP(pop = TS2, traits = 1, use = "pheno", snpChip =
1, simParam = SP)


# results C0
resultsC0 <-  data.frame(
  method = c("TRAD_SSD", "DRIFT_SSD", "SSD_GS_F2", "GS_F2",
"SDD_GS_F4", "SDD_GS_FS", "SDD_GS_FS_d"),
  Ne = n.parents,
  TS_size = c(rep(0, 2), rep(nInd(TS2), 5)),
  rep = 1,
  cycle = 0,
  G = meanG(pop.trad),
  Va = varA(pop.trad),
  BestG = max(gv(pop.trad)),
  Ac = sqrt(h2)
)

#####################################################
# simulating more cycles of breeding by SSD method
#####################################################
cat("SSD", "\n")

results.ssd <- foreach(k = r,
                       .packages = c("AlphaSimR"),
                       .combine = "rbind",
```

```
                       .multicombine = TRUE,
                       .errorhandling = "remove",
                       .verbose = TRUE
) %dopar% {

  pop.ssd <- pop.trad
  output <- data.frame()

  for (i in 1:cycles) {
    X0 <- meanG(pop.ssd)
    #F1 and F2
    f1 <- randCross(pop = pop.ssd, nCrosses = n.crosses, nProgeny =
1, simParam = SP)
    f2 <- self(pop = f1, nProgeny = progenie.size, simParam = SP)

    #F2 to F6 by SSD
    fn <- f2
    for (j in 1:4) {
      fn <- self(pop = fn, nProgeny = 1, simParam = SP)
    }

    # then select the best ones regardless the family
    newparents <- selectInd(pop = fn, nInd = n.parents, trait = 1,
"pheno", gender = "B", selectTop = TRUE, simParam = SP)
    pop.ssd <- newparents

    output <- rbind(output, data.frame(
      method = "TRAD_SSD",
      TS_size = 0,
      Ne = n.parents,
      rep = k,
      cycle = i,
      G = meanG(pop.ssd),
      Va = varA(pop.ssd),
      BestG = max(gv(pop.ssd)),
      Ac = sqrt(h2)
      ))
  }
  return(output)
}

#####################################################
# simulating more cycles of drift by SSD method
#####################################################
cat("DFRIT", "\n")

results.drift <- foreach(k = r,
                         .packages = c("AlphaSimR"),
                         .combine = "rbind",
                         .multicombine = TRUE,
                         .errorhandling = "remove",
                         .verbose = TRUE
) %dopar% {
```

```
  pop.drift <- pop.trad
  output <- data.frame()

  for (i in 1:cycles) {
    X0 <- meanG(pop.drift)
    #F1 and F2
    f1 <- randCross(pop = pop.drift, nCrosses = n.crosses, nProgeny
= 1, simParam = SP)
    f2 <- self(pop = f1, nProgeny = progenie.size, simParam = SP)

    #F2 to F6 by SSD
    fn <- f2
    for (j in 1:4) {
      fn <- self(pop = fn, nProgeny = 1, simParam = SP)
    }

    # then, select the best ones regardless the family
    newparents <- selectInd(pop = fn, nInd = n.parents, trait = 1,
"rand", gender = "B", selectTop = FALSE, simParam = SP)
    pop.drift <- newparents

    output <- rbind(output, data.frame(
      method = "DRIFT_SSD",
      TS_size = 0,
      Ne = n.parents,
      rep = k,
      cycle = i,
      G = meanG(pop.drift),
      Va = varA(pop.drift),
      BestG = max(gv(pop.drift)),
      Ac = 0
    ))
  }
  return(output)
}

#####################################################
# simulating more cycles of GS in F2 followed by the SSD
#####################################################
cat("GS_F2_SSD", "\n")

results.gs.ssd.f2 <- foreach(k = r,
                           .packages = c("AlphaSimR"),
                           .combine = "rbind",
                           .multicombine = TRUE,
                           .errorhandling = "remove",
                           .verbose = TRUE
) %dopar% {

  pop.gs <- pop.trad
  output <- data.frame()

  for (i in 1:cycles) {
    X0 <- meanG(pop.gs)
```

```
    #F1
    f1 <- randCross(pop = pop.gs, nCrosses = n.crosses, nProgeny =
1, simParam = SP)
    f2 <- self(pop = f1, nProgeny = progenie.size, simParam = SP)
    f2 <- setEBV(pop = f2, solution = markersC0, value = "bv",
simParam = SP)
    f2sel <- selectWithinFam(pop = f2, nInd = 1, trait = 1, use =
"ebv", gender = "B", simParam = SP)
    f3 <- self(pop = f2sel, nProgeny = progenie.size, simParam = SP)
    f4 <- self(pop = f3, nProgeny = 1, simParam = SP)

    # then select the best ones regardless the family
    newparents <- selectInd(pop = f4, nInd = n.parents, trait = 1,
"pheno", gender = "B", selectTop = TRUE, simParam = SP)
    pop.gs <- newparents

    output <- rbind(output, data.frame(
      method = "SSD_GS_F2",
      TS_size = nInd(TS2),
      Ne = n.parents,
      rep = k,
      cycle = i,
      G = meanG(pop.gs),
      Va = varA(pop.gs),
      BestG = max(gv(pop.gs)),
      Ac = cor(gv(f2), ebv(f2))
    ))

  }
  return(output)
}

#####################################################
# simulating more cycles of GS in F2 followed by recombination
#####################################################
cat("GS_F2", "\n")

results.gs.f2 <- foreach(k = r,
                             .packages = c("AlphaSimR"),
                             .combine = "rbind",
                             .multicombine = TRUE,
                             .errorhandling = "remove",
                             .verbose = TRUE
) %dopar% {

  pop.gs <- pop.trad
  output <- data.frame()

  for (i in 1:cycles) {
    X0 <- meanG(pop.gs)
    #F1
    f1 <- randCross(pop = pop.gs, nCrosses = n.crosses, nProgeny =
1, simParam = SP)
    f2 <- self(pop = f1, nProgeny = progenie.size, simParam = SP)
```

```
    f2 <- setEBV(pop = f2, solution = markersC0, value = "bv",
simParam = SP)

    # then select the best ones regardless the family
    newparents <- selectInd(pop = f2, nInd = n.parents, trait = 1,
"ebv", gender = "B", selectTop = TRUE, simParam = SP)
    pop.gs <- newparents

    output <- rbind(output, data.frame(
      method = "GS_F2",
      TS_size = nInd(TS2),
      Ne = n.parents,
      rep = k,
      cycle = i,
      G = meanG(pop.gs),
      Va = varA(pop.gs),
      BestG = max(gv(pop.gs)),
      Ac = cor(gv(f2), ebv(f2))
    ))

  }
  return(output)
}

#####################################################
# simulating more cycles of GS in F4 followed by the SSD
#####################################################
cat("GS_SSDGSF4", "\n")

results.gs.ssd.f4 <- foreach(k = r,
                         .packages = c("AlphaSimR"),
                         .combine = "rbind",
                         .multicombine = TRUE,
                         .errorhandling = "remove",
                         .verbose = TRUE
) %dopar% {

  pop.gs <- pop.trad
  output <- data.frame()

  for (i in 1:cycles) {
    X0 <- meanG(pop.gs)
    #F1
    f1 <- randCross(pop = pop.gs, nCrosses = n.crosses, nProgeny =
1, simParam = SP)
    f2 <- self(pop = f1, nProgeny = progenie.size, simParam = SP)

    #F2 to F4 by SSD
    fn <- f2
    for (j in 1:2) {
      fn <- self(pop = fn, nProgeny = 1, simParam = SP)
    }

    #F4
```

```
    f4 <- fn

    #then, apply GS
    f4 <- setEBV(pop = f4, solution = markersC0, value = "bv",
simParam = SP)

    # select the best ones regardless the family
    newparents <- selectInd(pop = f4, nInd = n.parents, trait = 1,
use = "ebv", gender = "B", simParam = SP)

    pop.gs <- newparents

    output <- rbind(output, data.frame(
      method = "SDD_GS_F4",
      TS_size = nInd(TS2),
      Ne = n.parents,
      rep = k,
      cycle = i,
      G = meanG(pop.gs),
      Va = varA(pop.gs),
      BestG = max(gv(pop.gs)),
      Ac = cor(gv(f4), ebv(f4))
    ))

  }
  return(output)
}

#################################
# more cycles of GS + FS selecting the two best in F2
################################
cat("SDD_GS_FS", "\n")

results.gs.fs <- foreach(k = r,
                            .packages = c("AlphaSimR"),
                            .combine = "rbind",
                            .multicombine = TRUE,
                            .errorhandling = "remove",
                            .verbose = TRUE
) %dopar% {

  pop.gsfs <- pop.trad
  output <- data.frame()

  for (i in 1:cycles) {
    cat("Processing the cycle", i, "\n")
    X0 <- meanG(pop.gsfs)
    #F1
    f1 <- randCross(pop = pop.gsfs, nCrosses = n.crosses, nProgeny =
1, simParam = SP)
    f2 <- self(pop = f1, nProgeny = progenie.size, simParam = SP)
    f2 <- setEBV(pop = f2, solution = markersC0, value = "bv",
simParam = SP)
    f2sel <- selectWithinFam(pop = f2, nInd = 2, trait = 1, use =
```

```
"ebv", gender = "B", simParam = SP)
    crossPlan <- matrix(1:length(f2sel@id), nrow = n.crosses, ncol =
2, byrow = TRUE)
    f1seg <- makeCross(pop = f2sel, crossPlan = crossPlan, simParam
= SP)
    f2new <- self(pop = f1seg, nProgeny = progenie.size, simParam =
SP)

    #F2new to F4 by SSD
    fn <- f2new
    for (j in 1:2) {
      fn <- self(pop = fn, nProgeny = 1, simParam = SP)
    }

    # then select the best ones regardless the family
    newparents <- selectInd(pop = fn, nInd = n.parents, trait = 1,
"pheno", gender = "B", selectTop = TRUE, simParam = SP)
    pop.gsfs <- newparents

    output <- rbind(output, data.frame(
      method = "SDD_GS_FS",
      TS_size = nInd(TS2),
      Ne = n.parents,
      rep = k,
      cycle = i,
      G = meanG(pop.gsfs),
      Va = varA(pop.gsfs),
      BestG = max(gv(pop.gsfs)),
      Ac = cor(gv(f2), ebv(f2))
    ))

  }
  return(output)
}


#######################################################
# more cycles of GS + FS selecting two good and distant sibs (IS =
10%)
#######################################################
cat("SDD_GS_FS_d", "\n")

results.gs.fs.d <- foreach(k = r,
                            .packages = c("AlphaSimR"),
                            .combine = "rbind",
                            .multicombine = TRUE,
                            .errorhandling = "remove",
                            .verbose = TRUE
) %dopar% {

  pop.gsfsd <- pop.trad
  output <- data.frame()

  for (i in 1:cycles) {
```

```
    X0 <- meanG(pop.gsfsd)
    #F1
    f1 <- randCross(pop = pop.gsfsd, nCrosses = n.crosses, nProgeny
= 1, simParam = SP)
    f2 <- self(pop = f1, nProgeny = progenie.size, simParam = SP)
    f2 <- setEBV(pop = f2, solution = markersC0, value = "bv",
simParam = SP)
    f2sel <- selectWithinFam(pop = f2, nInd = progenie.size * 0.1,
trait = 1, use = "ebv", gender = "B", simParam = SP)
    chipteste <- pullSnpGeno(pop = f2sel, snpChip = 1, simParam =
NULL)
    ibs <- as.matrix(dist(x = chipteste, method = "euclidean", diag
= T, upper = T, p = 2))

    # after the selection per family, it is time to identify those
most distant sibs in order to cross them
    parents <- c()

    for (p in 1 : n.crosses) {
      ibsfam <- ibs[(((p - 1) * 2) + 1):(p * 2), (((p - 1) * 2) +
1):(p * 2)]
      parents <- c(parents, c(rownames(which(ibsfam == max(ibsfam),
arr.ind = TRUE)))[1:2])
    }

    crossPlan <- matrix(match(parents, colnames(ibs)), nrow =
n.crosses, ncol = 2, byrow = TRUE)
    f1seg <- makeCross(pop = f2sel, crossPlan = crossPlan, simParam
= SP)
    f2new <- self(pop = f1seg, nProgeny = progenie.size, simParam =
SP)

    #F2new to F4 by SSD
    fn <- f2new
    for (j in 1:2) {
      fn <- self(pop = fn, nProgeny = 1, simParam = SP)
    }

    # then select the best one per family
    newparents <- selectInd(pop = fn, nInd = n.parents, trait = 1,
"pheno", gender = "B", selectTop = TRUE, simParam = SP)
    pop.gsfsd <- newparents

    output <- rbind(output, data.frame(
      method = "SDD_GS_FS_d",
      TS_size = nInd(TS2),
      Ne = n.parents,
      rep = k,
      cycle = i,
      G = meanG(pop.gsfsd),
      Va = varA(pop.gsfsd),
      BestG = max(gv(pop.gsfsd)),
      Ac = cor(gv(f2), ebv(f2))
    ))
```

```
  }
  return(output)
}

########### saving the outputs
cat("saving the output 1", "\n")
output <- rbind(resultsC0, results.ssd, results.drift,
results.gs.ssd.f2, results.gs.f2, results.gs.ssd.f4, results.gs.fs,
results.gs.fs.d)
saveRDS(output, paste("output", n.parents, sep = ""))


#######################################################
############## updating TS scenarios #################
#######################################################

resultsC0 <-  data.frame(
  method = c("TRAD_SSD", "DRIFT_SSD", "SSD_GS_F2", "SSD_GS_TScn",
"SSD_GS_TSall", "SSD_GS_TSgpo"),
  Ne = n.parents,
  TS_size = c(0, 0, 1536, 384, 384, 384),
  rep = 1,
  cycle = 0,
  G = meanG(pop.trad),
  Va = varA(pop.trad),
  BestG = max(gv(pop.trad)),
  Ac = sqrt(h2)
)

#####################################################
# simulating more cycles of GS
# where a new TS of 384 is create inside each cycle
#####################################################
cat("GS_TSCn", "\n")

results.gs.tscn <- foreach(k = r,
                           .packages = c("AlphaSimR"),
                           .combine = "rbind",
                           .multicombine = TRUE,
                           .errorhandling = "remove",
                           .verbose = TRUE
) %dopar% {

  pop.gs <- pop.trad
  output <- data.frame()
  TS <- TS000
  snps <- markers

  for (i in 1:cycles) {
    X0 <- meanG(pop.gs)
    #F1
    f1 <- randCross(pop = pop.gs, nCrosses = n.crosses, nProgeny =
1, simParam = SP)
```

```
    f2 <- self(pop = f1, nProgeny = progenie.size, simParam = SP)
    f2 <- setEBV(pop = f2, solution = snps, value = "bv", simParam =
SP)
    f2sel <- selectWithinFam(pop = f2, nInd = 1, trait = 1, use =
"ebv", gender = "B", simParam = SP)
    f3 <- self(pop = f2sel, nProgeny = progenie.size, simParam = SP)
    f4 <- self(pop = f3, nProgeny = 1, simParam = SP)

    # then select the best ones regardless the family
    newparents <- selectInd(pop = f4, nInd = n.parents, trait = 1,
"pheno", gender = "B", selectTop = TRUE, simParam = SP)
    pop.gs <- newparents

    output <- rbind(output, data.frame(
      method = "SSD_GS_TScn",
      TS_size = nInd(TS),
      Ne = n.parents,
      rep = k,
      cycle = i,
      G = meanG(pop.gs),
      Va = varA(pop.gs),
      BestG = max(gv(pop.gs)),
      Ac = cor(gv(f2), ebv(f2))
    ))

    # updating the TS and marker effects
    TS <- selectWithinFam(pop = f4, nInd = 13, trait = 1, use =
"rand", gender = "B", simParam = SP)[1:384]
    snps <- RRBLUP(pop = TS, traits = 1, use = "pheno", snpChip = 1,
simParam = SP)

  }
  return(output)
}

#####################################################
# simulating more cycles of GS
# where a new set of 384 is added to TS each cycle
#####################################################
cat("GS_TSCall", "\n")

results.gs.tsall <- foreach(k = r,
                            .packages = c("AlphaSimR"),
                            .combine = "rbind",
                            .multicombine = TRUE,
                            .errorhandling = "remove",
                            .verbose = TRUE
) %dopar% {

  pop.gs <- pop.trad
  output <- data.frame()
  TS <- TS000
  snps <- markers
```

```
  for (i in 1:cycles) {
    X0 <- meanG(pop.gs)
    #F1
    f1 <- randCross(pop = pop.gs, nCrosses = n.crosses, nProgeny =
1, simParam = SP)
    f2 <- self(pop = f1, nProgeny = progenie.size, simParam = SP)
    f2 <- setEBV(pop = f2, solution = snps, value = "bv", simParam =
SP)
    f2sel <- selectWithinFam(pop = f2, nInd = 1, trait = 1, use =
"ebv", gender = "B", simParam = SP)
    f3 <- self(pop = f2sel, nProgeny = progenie.size, simParam = SP)
    f4 <- self(pop = f3, nProgeny = 1, simParam = SP)

    # then select the best ones regardless the family
    newparents <- selectInd(pop = f4, nInd = n.parents, trait = 1,
"pheno", gender = "B", selectTop = TRUE, simParam = SP)
    pop.gs <- newparents


    output <- rbind(output, data.frame(
      method = "SSD_GS_TSall",
      TS_size = nInd(TS),
      Ne = n.parents,
      rep = k,
      cycle = i,
      G = meanG(pop.gs),
      Va = varA(pop.gs),
      BestG = max(gv(pop.gs)),
      Ac = cor(gv(f2), ebv(f2))
    ))

    # updating the TS and marker effects
    TS2add <- selectWithinFam(pop = f4, nInd = 13, trait = 1, use =
"rand", gender = "B", simParam = SP)[1:384]
    popList <- list(TS, TS2add)
    TS <- mergePops(popList)
    snps <- RRBLUP2(pop = TS, traits = 1, use = "pheno", snpChip =
1, simParam = SP)
  }
  return(output)
}

#####################################################
# simulating more cycles of GS
# where a new group is added in the each cycle until we have 3
generations
# then, always the oldest one is eliminated
#####################################################
cat("GS_TSgpo", "\n")

results.gs.tsgpo <- foreach(k = r,
                            .packages = c("AlphaSimR"),
                            .combine = "rbind",
                            .multicombine = TRUE,
```

```
                            .errorhandling = "remove",
                            .verbose = TRUE
) %dopar% {

  pop.gs <- pop.trad
  output <- data.frame()
  TS <- TS000
  popList <- list(TS)
  snps <- markers

  for (i in 1:cycles) {
    X0 <- meanG(pop.gs)
    #F1
    f1 <- randCross(pop = pop.gs, nCrosses = n.crosses, nProgeny =
1, simParam = SP)
    f2 <- self(pop = f1, nProgeny = progenie.size, simParam = SP)
    f2 <- setEBV(pop = f2, solution = snps, value = "bv", simParam =
SP)
    f2sel <- selectWithinFam(pop = f2, nInd = 1, trait = 1, use =
"ebv", gender = "B", simParam = SP)
    f3 <- self(pop = f2sel, nProgeny = progenie.size, simParam = SP)
    f4 <- self(pop = f3, nProgeny = 1, simParam = SP)

    # then select the best ones regardless the family
    newparents <- selectInd(pop = f4, nInd = n.parents, trait = 1,
"pheno", gender = "B", selectTop = TRUE, simParam = SP)
    pop.gs <- newparents


    output <- rbind(output, data.frame(
      method = "SSD_GS_TSgpo",
      TS_size = nInd(TS),
      Ne = n.parents,
      rep = k,
      cycle = i,
      G = meanG(pop.gs),
      Va = varA(pop.gs),
      BestG = max(gv(pop.gs)),
      Ac = cor(gv(f2), ebv(f2))
    ))

    # updating the TS and marker effects
    TSi <- selectWithinFam(pop = f4, nInd = 13, trait = 1, use =
"rand", gender = "B", simParam = SP)[1:384]
    popList <- c(popList, TSi)

    if(length(popList) <= 3) {
      TS <- mergePops(popList)
    }

    if(length(popList) > 3) {
      TS <- mergePops(popList[(length(popList)-2):length(popList)])
    }
```

```
    snps <- RRBLUP(pop = TS, traits = 1, use = "pheno", snpChip = 1,
simParam = SP)

  }

  return(output)
}

########### saving the outputs
cat("saving the output 2", "\n")
output <- rbind(resultsC0, results.ssd, results.drift,
results.gs.ssd.f2, results.gs.tscn, results.gs.tsall,
results.gs.tsgpo)
saveRDS(output, paste("output", "TS", sep = ""))

####################################################################
##################
#################### simulating more cycles of GS, updating sizes
###################
####################################################################
##################

resultsC0 <-  data.frame(
  method = c("TRAD_SSD", "DRIFT_SSD", "TS_0", "TS_384", "TS_768",
"TS_1152"),
  Ne = n.parents,
  TS_size = c(0, 0, 384, 384, 384, 384),
  rep = 1,
  cycle = 0,
  G = meanG(pop.trad),
  Va = varA(pop.trad),
  BestG = max(gv(pop.trad)),
  Ac = sqrt(h2)
)


#####################################################
# simulating more cycles of GS updating GPO
# where a new set is added to TS each cycle, varying the size
#####################################################

###############################
# 0 will be added every cycle
###############################
cat("TS_0", "\n")

results.ts.0 <- foreach(k = r,
                            .packages = c("AlphaSimR"),
                            .combine = "rbind",
                            .multicombine = TRUE,
                            .errorhandling = "remove",
                            .verbose = TRUE
) %dopar% {
```

```
  pop.gs <- pop.trad
  output <- data.frame()

  for (i in 1:cycles) {
    X0 <- meanG(pop.gs)
    #F1
    f1 <- randCross(pop = pop.gs, nCrosses = n.crosses, nProgeny =
1, simParam = SP)
    f2 <- self(pop = f1, nProgeny = progenie.size, simParam = SP)
    f2 <- setEBV(pop = f2, solution = markers, value = "bv",
simParam = SP)
    f2sel <- selectWithinFam(pop = f2, nInd = 1, trait = 1, use =
"ebv", gender = "B", simParam = SP)
    f3 <- self(pop = f2sel, nProgeny = progenie.size, simParam = SP)
    f4 <- self(pop = f3, nProgeny = 1, simParam = SP)

    # then select the best ones regardless the family
    newparents <- selectInd(pop = f4, nInd = n.parents, trait = 1,
"pheno", gender = "B", selectTop = TRUE, simParam = SP)
    pop.gs <- newparents

    output <- rbind(output, data.frame(
      method = "TS_0",
      TS_size = nInd(TS000),
      Ne = n.parents,
      rep = k,
      cycle = i,
      G = meanG(pop.gs),
      Va = varA(pop.gs),
      BestG = max(gv(pop.gs)),
      Ac = cor(gv(f2), ebv(f2))
    ))

  }
  return(output)
}

###############################
# 384 will be added every cycle
###############################
cat("TS_384", "\n")

results.ts.384 <- foreach(k = r,
                          .packages = c("AlphaSimR"),
                          .combine = "rbind",
                          .multicombine = TRUE,
                          .errorhandling = "remove",
                          .verbose = TRUE
) %dopar% {

  pop.gs <- pop.trad
  output <- data.frame()
  TS <- TS000
  popList <- list(TS)
```

```
  snps <- markers


  for (i in 1:cycles) {
    X0 <- meanG(pop.gs)
    #F1
    f1 <- randCross(pop = pop.gs, nCrosses = n.crosses, nProgeny =
1, simParam = SP)
    f2 <- self(pop = f1, nProgeny = progenie.size, simParam = SP)
    f2 <- setEBV(pop = f2, solution = snps, value = "bv", simParam =
SP)
    f2sel <- selectWithinFam(pop = f2, nInd = 1, trait = 1, use =
"ebv", gender = "B", simParam = SP)
    f3 <- self(pop = f2sel, nProgeny = progenie.size, simParam = SP)
    f4 <- self(pop = f3, nProgeny = 1, simParam = SP)

    # then select the best ones regardless the family
    newparents <- selectInd(pop = f4, nInd = n.parents, trait = 1,
"pheno", gender = "B", selectTop = TRUE, simParam = SP)
    pop.gs <- newparents

    output <- rbind(output, data.frame(
      method = "TS_384",
      TS_size = nInd(TS),
      Ne = n.parents,
      rep = k,
      cycle = i,
      G = meanG(pop.gs),
      Va = varA(pop.gs),
      BestG = max(gv(pop.gs)),
      Ac = cor(gv(f2), ebv(f2))
    ))

    # updating the TS and marker effects
    TSi <- selectWithinFam(pop = f4, nInd = 13, trait = 1, use =
"rand", gender = "B", simParam = SP)[1:384]
    popList <- c(popList, TSi)

    if(length(popList) <= 3) {
      TS <- mergePops(popList)}

    if(length(popList) > 3) {
      TS <- mergePops(popList[(length(popList)-2):length(popList)])}

    snps <- RRBLUP2(pop = TS, traits = 1, use = "pheno", snpChip =
1, simParam = SP)
  }

  return(output)
}

#####################################################
# where a new set of 768 is added to TS each cycle
#####################################################
```

```
cat("TS_768", "\n")

results.ts.768 <- foreach(k = r,
                          .packages = c("AlphaSimR"),
                          .combine = "rbind",
                          .multicombine = TRUE,
                          .errorhandling = "remove",
                          .verbose = TRUE
) %dopar% {

  pop.gs <- pop.trad
  output <- data.frame()
  TS <- TS000
  popList <- list(TS)
  snps <- markers

  for (i in 1:cycles) {
    X0 <- meanG(pop.gs)
    #F1
    f1 <- randCross(pop = pop.gs, nCrosses = n.crosses, nProgeny =
1, simParam = SP)
    f2 <- self(pop = f1, nProgeny = progenie.size, simParam = SP)
    f2 <- setEBV(pop = f2, solution = snps, value = "bv", simParam =
SP)
    f2sel <- selectWithinFam(pop = f2, nInd = 1, trait = 1, use =
"ebv", gender = "B", simParam = SP)
    f3 <- self(pop = f2sel, nProgeny = progenie.size, simParam = SP)
    f4 <- self(pop = f3, nProgeny = 1, simParam = SP)

    # then select the best ones regardless the family
    newparents <- selectInd(pop = f4, nInd = n.parents, trait = 1,
"pheno", gender = "B", selectTop = TRUE, simParam = SP)
    pop.gs <- newparents


    output <- rbind(output, data.frame(
      method = "TS_768",
      TS_size = nInd(TS),
      Ne = n.parents,
      rep = k,
      cycle = i,
      G = meanG(pop.gs),
      Va = varA(pop.gs),
      BestG = max(gv(pop.gs)),
      Ac = cor(gv(f2), ebv(f2))
    ))

    # updating the TS and marker effects
    TSi <- selectWithinFam(pop = f4, nInd = 26, trait = 1, use =
"rand", gender = "B", simParam = SP)[1:768]
    popList <- c(popList, TSi)

    if(length(popList) <= 3) {
      TS <- mergePops(popList)}
```

```
    if(length(popList) > 3) {
      TS <- mergePops(popList[(length(popList)-2):length(popList)])}

    snps <- RRBLUP2(pop = TS, traits = 1, use = "pheno", snpChip =
1, simParam = SP)
  }

  return(output)
}


#####################################################
# where a new set of 1152 is added to TS each cycle
#####################################################
cat("TS_1152", "\n")

results.ts.1152 <- foreach(k = r,
                           .packages = c("AlphaSimR"),
                           .combine = "rbind",
                           .multicombine = TRUE,
                           .errorhandling = "remove",
                           .verbose = TRUE
) %dopar% {

  pop.gs <- pop.trad
  output <- data.frame()
  TS <- TS000
  popList <- list(TS)
  snps <- markers

  for (i in 1:cycles) {
    X0 <- meanG(pop.gs)
    #F1
    f1 <- randCross(pop = pop.gs, nCrosses = n.crosses, nProgeny =
1, simParam = SP)
    f2 <- self(pop = f1, nProgeny = progenie.size, simParam = SP)
    f2 <- setEBV(pop = f2, solution = snps, value = "bv", simParam =
SP)
    f2sel <- selectWithinFam(pop = f2, nInd = 1, trait = 1, use =
"ebv", gender = "B", simParam = SP)
    f3 <- self(pop = f2sel, nProgeny = progenie.size, simParam = SP)
    f4 <- self(pop = f3, nProgeny = 1, simParam = SP)

    # then select the best ones regardless the family
    newparents <- selectInd(pop = f4, nInd = n.parents, trait = 1,
"pheno", gender = "B", selectTop = TRUE, simParam = SP)
    pop.gs <- newparents

    output <- rbind(output, data.frame(
      method = "TS_1152",
      TS_size = nInd(TS),
      Ne = n.parents,
      rep = k,
```

```
      cycle = i,
      G = meanG(pop.gs),
      Va = varA(pop.gs),
      BestG = max(gv(pop.gs)),
      Ac = cor(gv(f2), ebv(f2))
    ))

    # updating the TS and marker effects
    TSi <- selectWithinFam(pop = f4, nInd = 39, trait = 1, use =
"rand", gender = "B", simParam = SP)[1:1152]
    popList <- c(popList, TSi)

    if(length(popList) <= 3) {
      TS <- mergePops(popList)}

    if(length(popList) > 3) {
      TS <- mergePops(popList[(length(popList)-2):length(popList)])}

    snps <- RRBLUP2(pop = TS, traits = 1, use = "pheno", snpChip =
1, simParam = SP)

  }

  return(output)
}

########### saving the outputs
cat("saving the output TS size", "\n")
output <- rbind(resultsC0, results.ssd, results.drift, results.ts.0,
results.ts.384, results.ts.768, results.ts.1152)
saveRDS(output, paste("output_", "TS_sizes", sep = ""))


####################################################################
######
############ returning to breeding schemes but with 24 parents
###########
####################################################################
######
n.parents <- 24

resultsC0 <-  data.frame(
  method = c("TRAD_SSD", "DRIFT_SSD", "SSD_GS_F2", "GS_F2",
"SDD_GS_F4", "SDD_GS_FS", "SDD_GS_FS_d"),
  Ne = n.parents,
  TS_size = c(rep(0, 2), rep(nInd(TS2), 5)),
  rep = 1,
  cycle = 0,
  G = meanG(pop.trad),
  Va = varA(pop.trad),
  BestG = max(gv(pop.trad)),
  Ac = sqrt(h2)
)
```

```
######################################################
# simulating more cycles of breeding by SSD method
######################################################
cat("SSD", "\n")

results.ssd <- foreach(k = r,
                       .packages = c("AlphaSimR"),
                       .combine = "rbind",
                       .multicombine = TRUE,
                       .errorhandling = "remove",
                       .verbose = TRUE
) %dopar% {

  pop.ssd <- pop.trad
  output <- data.frame()

  for (i in 1:cycles) {
    X0 <- meanG(pop.ssd)
    #F1 and F2
    f1 <- randCross(pop = pop.ssd, nCrosses = n.crosses, nProgeny =
1, simParam = SP)
    f2 <- self(pop = f1, nProgeny = progenie.size, simParam = SP)

    #F2 to F6 by SSD
    fn <- f2
    for (j in 1:4) {
      fn <- self(pop = fn, nProgeny = 1, simParam = SP)
    }

    # then select the best ones regardless the family
    newparents <- selectInd(pop = fn, nInd = n.parents, trait = 1,
"pheno", gender = "B", selectTop = TRUE, simParam = SP)
    pop.ssd <- newparents

    output <- rbind(output, data.frame(
      method = "TRAD_SSD",
      TS_size = 0,
      Ne = n.parents,
      rep = k,
      cycle = i,
      G = meanG(pop.ssd),
      Va = varA(pop.ssd),
      BestG = max(gv(pop.ssd)),
      Ac = sqrt(h2)
    ))
  }
  return(output)
}

######################################################
# simulating more cycles of drift by SSD method
######################################################
cat("DFRIT", "\n")
```

```
results.drift <- foreach(k = r,
                         .packages = c("AlphaSimR"),
                         .combine = "rbind",
                         .multicombine = TRUE,
                         .errorhandling = "remove",
                         .verbose = TRUE
) %dopar% {

  pop.drift <- pop.trad
  output <- data.frame()

  for (i in 1:cycles) {
    X0 <- meanG(pop.drift)
    #F1 and F2
    f1 <- randCross(pop = pop.drift, nCrosses = n.crosses, nProgeny
= 1, simParam = SP)
    f2 <- self(pop = f1, nProgeny = progenie.size, simParam = SP)

    #F2 to F6 by SSD
    fn <- f2
    for (j in 1:4) {
      fn <- self(pop = fn, nProgeny = 1, simParam = SP)
    }

    # then, select the best ones regardless the family
    newparents <- selectInd(pop = fn, nInd = n.parents, trait = 1,
"rand", gender = "B", selectTop = FALSE, simParam = SP)
    pop.drift <- newparents

    output <- rbind(output, data.frame(
      method = "DRIFT_SSD",
      TS_size = 0,
      Ne = n.parents,
      rep = k,
      cycle = i,
      G = meanG(pop.drift),
      Va = varA(pop.drift),
      BestG = max(gv(pop.drift)),
      Ac = 0
    ))
  }
  return(output)
}

#####################################################
# simulating more cycles of GS in F2 followed by the SSD
#####################################################
cat("GS_F2_SSD", "\n")

results.gs.ssd.f2 <- foreach(k = r,
                             .packages = c("AlphaSimR"),
                             .combine = "rbind",
                             .multicombine = TRUE,
                             .errorhandling = "remove",
```

```
                            .verbose = TRUE
) %dopar% {

  pop.gs <- pop.trad
  output <- data.frame()

  for (i in 1:cycles) {
    X0 <- meanG(pop.gs)
    #F1
    f1 <- randCross(pop = pop.gs, nCrosses = n.crosses, nProgeny =
1, simParam = SP)
    f2 <- self(pop = f1, nProgeny = progenie.size, simParam = SP)
    f2 <- setEBV(pop = f2, solution = markersC0, value = "bv",
simParam = SP)
    f2sel <- selectWithinFam(pop = f2, nInd = 1, trait = 1, use =
"ebv", gender = "B", simParam = SP)
    f3 <- self(pop = f2sel, nProgeny = progenie.size, simParam = SP)
    f4 <- self(pop = f3, nProgeny = 1, simParam = SP)

    # then select the best ones regardless the family
    newparents <- selectInd(pop = f4, nInd = n.parents, trait = 1,
"pheno", gender = "B", selectTop = TRUE, simParam = SP)
    pop.gs <- newparents

    output <- rbind(output, data.frame(
      method = "SSD_GS_F2",
      TS_size = nInd(TS2),
      Ne = n.parents,
      rep = k,
      cycle = i,
      G = meanG(pop.gs),
      Va = varA(pop.gs),
      BestG = max(gv(pop.gs)),
      Ac = cor(gv(f2), ebv(f2))
    ))

  }
  return(output)
}



#####################################################
# simulating more cycles of GS in F2 followed by recombination
#####################################################
cat("GS_F2", "\n")

results.gs.f2 <- foreach(k = r,
                         .packages = c("AlphaSimR"),
                         .combine = "rbind",
                         .multicombine = TRUE,
                         .errorhandling = "remove",
                         .verbose = TRUE
) %dopar% {
```

```
  pop.gs <- pop.trad
  output <- data.frame()

  for (i in 1:cycles) {
    X0 <- meanG(pop.gs)
    #F1
    f1 <- randCross(pop = pop.gs, nCrosses = n.crosses, nProgeny =
1, simParam = SP)
    f2 <- self(pop = f1, nProgeny = progenie.size, simParam = SP)
    f2 <- setEBV(pop = f2, solution = markersC0, value = "bv",
simParam = SP)

    # then select the best ones regardless the family
    newparents <- selectInd(pop = f2, nInd = n.parents, trait = 1,
"ebv", gender = "B", selectTop = TRUE, simParam = SP)
    pop.gs <- newparents

    output <- rbind(output, data.frame(
      method = "GS_F2",
      TS_size = nInd(TS2),
      Ne = n.parents,
      rep = k,
      cycle = i,
      G = meanG(pop.gs),
      Va = varA(pop.gs),
      BestG = max(gv(pop.gs)),
      Ac = cor(gv(f2), ebv(f2))
    ))

  }
  return(output)
}

#####################################################
# simulating more cycles of GS in F4 followed by the SSD
#####################################################
cat("GS_SSDGSF4", "\n")

results.gs.ssd.f4 <- foreach(k = r,
                             .packages = c("AlphaSimR"),
                             .combine = "rbind",
                             .multicombine = TRUE,
                             .errorhandling = "remove",
                             .verbose = TRUE
) %dopar% {

  pop.gs <- pop.trad
  output <- data.frame()

  for (i in 1:cycles) {
    X0 <- meanG(pop.gs)
    #F1
    f1 <- randCross(pop = pop.gs, nCrosses = n.crosses, nProgeny =
```

```
1, simParam = SP)
    f2 <- self(pop = f1, nProgeny = progenie.size, simParam = SP)

    #F2 to F4 by SSD
    fn <- f2
    for (j in 1:2) {
      fn <- self(pop = fn, nProgeny = 1, simParam = SP)
    }

    #F4
    f4 <- fn

    #then, apply GS
    f4 <- setEBV(pop = f4, solution = markersC0, value = "bv",
simParam = SP)

    # select the best ones regardless the family
    newparents <- selectInd(pop = f4, nInd = n.parents, trait = 1,
use = "ebv", gender = "B", simParam = SP)

    pop.gs <- newparents

    output <- rbind(output, data.frame(
      method = "SDD_GS_F4",
      TS_size = nInd(TS2),
      Ne = n.parents,
      rep = k,
      cycle = i,
      G = meanG(pop.gs),
      Va = varA(pop.gs),
      BestG = max(gv(pop.gs)),
      Ac = cor(gv(f4), ebv(f4))
    ))

  }
  return(output)
}

#################################
# more cycles of GS + FS selecting the two best in F2
################################
cat("SDD_GS_FS", "\n")

results.gs.fs <- foreach(k = r,
                         .packages = c("AlphaSimR"),
                         .combine = "rbind",
                         .multicombine = TRUE,
                         .errorhandling = "remove",
                         .verbose = TRUE
) %dopar% {

  pop.gsfs <- pop.trad
  output <- data.frame()
```

```
  for (i in 1:cycles) {
    cat("Processing the cycle", i, "\n")
    X0 <- meanG(pop.gsfs)
    #F1
    f1 <- randCross(pop = pop.gsfs, nCrosses = n.crosses, nProgeny =
1, simParam = SP)
    f2 <- self(pop = f1, nProgeny = progenie.size, simParam = SP)
    f2 <- setEBV(pop = f2, solution = markersC0, value = "bv",
simParam = SP)
    f2sel <- selectWithinFam(pop = f2, nInd = 2, trait = 1, use =
"ebv", gender = "B", simParam = SP)
    crossPlan <- matrix(1:length(f2sel@id), nrow = n.crosses, ncol =
2, byrow = TRUE)
    f1seg <- makeCross(pop = f2sel, crossPlan = crossPlan, simParam
= SP)
    f2new <- self(pop = f1seg, nProgeny = progenie.size, simParam =
SP)

    #F2new to F4 by SSD
    fn <- f2new
    for (j in 1:2) {
      fn <- self(pop = fn, nProgeny = 1, simParam = SP)
    }

    # then select the best ones regardless the family
    newparents <- selectInd(pop = fn, nInd = n.parents, trait = 1,
"pheno", gender = "B", selectTop = TRUE, simParam = SP)
    pop.gsfs <- newparents

    output <- rbind(output, data.frame(
      method = "SDD_GS_FS",
      TS_size = nInd(TS2),
      Ne = n.parents,
      rep = k,
      cycle = i,
      G = meanG(pop.gsfs),
      Va = varA(pop.gsfs),
      BestG = max(gv(pop.gsfs)),
      Ac = cor(gv(f2), ebv(f2))
    ))

  }
  return(output)
}

#######################################################
# more cycles of GS + FS selecting two good and distant sibs (IS =
10%)
#######################################################
cat("SDD_GS_FS_d", "\n")

results.gs.fs.d <- foreach(k = r,
                           .packages = c("AlphaSimR"),
                           .combine = "rbind",
```

```
                           .multicombine = TRUE,
                           .errorhandling = "remove",
                           .verbose = TRUE
) %dopar% {

  pop.gsfsd <- pop.trad
  output <- data.frame()

  for (i in 1:cycles) {
    X0 <- meanG(pop.gsfsd)
    #F1
    f1 <- randCross(pop = pop.gsfsd, nCrosses = n.crosses, nProgeny
= 1, simParam = SP)
    f2 <- self(pop = f1, nProgeny = progenie.size, simParam = SP)
    f2 <- setEBV(pop = f2, solution = markersC0, value = "bv",
simParam = SP)
    f2sel <- selectWithinFam(pop = f2, nInd = progenie.size * 0.1,
trait = 1, use = "ebv", gender = "B", simParam = SP)
    chipteste <- pullSnpGeno(pop = f2sel, snpChip = 1, simParam =
NULL)
    ibs <- as.matrix(dist(x = chipteste, method = "euclidean", diag
= T, upper = T, p = 2))

    # after the selection per family, it is time to identify those
most distant sibs in order to cross them
    parents <- c()

    for (p in 1 : n.crosses) {
      ibsfam <- ibs[(((p - 1) * 2) + 1):(p * 2), (((p - 1) * 2) +
1):(p * 2)]
      parents <- c(parents, c(rownames(which(ibsfam == max(ibsfam),
arr.ind = TRUE)))[1:2])
    }

    crossPlan <- matrix(match(parents, colnames(ibs)), nrow =
n.crosses, ncol = 2, byrow = TRUE)
    f1seg <- makeCross(pop = f2sel, crossPlan = crossPlan, simParam
= SP)
    f2new <- self(pop = f1seg, nProgeny = progenie.size, simParam =
SP)

    #F2new to F4 by SSD
    fn <- f2new
    for (j in 1:2) {
      fn <- self(pop = fn, nProgeny = 1, simParam = SP)
    }

    # then select the best one per family
    newparents <- selectInd(pop = fn, nInd = n.parents, trait = 1,
"pheno", gender = "B", selectTop = TRUE, simParam = SP)
    pop.gsfsd <- newparents

    output <- rbind(output, data.frame(
      method = "SDD_GS_FS_d",
```

```
      TS_size = nInd(TS2),
      Ne = n.parents,
      rep = k,
      cycle = i,
      G = meanG(pop.gsfsd),
      Va = varA(pop.gsfsd),
      BestG = max(gv(pop.gsfsd)),
      Ac = cor(gv(f2), ebv(f2))
    ))

  }
  return(output)
}

########### saving the outputs
cat("saving the output 3", "\n")
output <- rbind(resultsC0, results.ssd, results.drift,
results.gs.ssd.f2, results.gs.f2, results.gs.ssd.f4, results.gs.fs,
results.gs.fs.d)
saveRDS(output, paste("output", n.parents, sep = ""))
#####################################################
stopImplicitCluster()
(end.time <- Sys.time())
(start.time - end.time)
############ the end ############
```
